# Supplementary material for: The Maize Clade A PP2C Phosphatases Play Critical Roles in Multiple Abiotic Stress Responses
Source: Int J Mol Sci. 2019 Jul 22;20(14):3573. doi: 10.3390/ijms20143573 (PMC6679055; doi:10.3390/ijms20143573)
Supplement: Supplementary file 1 [file ijms-20-03573-s001.zip › supplementary files of 2019.7.22 ijms 553347/Supplementary File(s).pptx]

## Slide 1
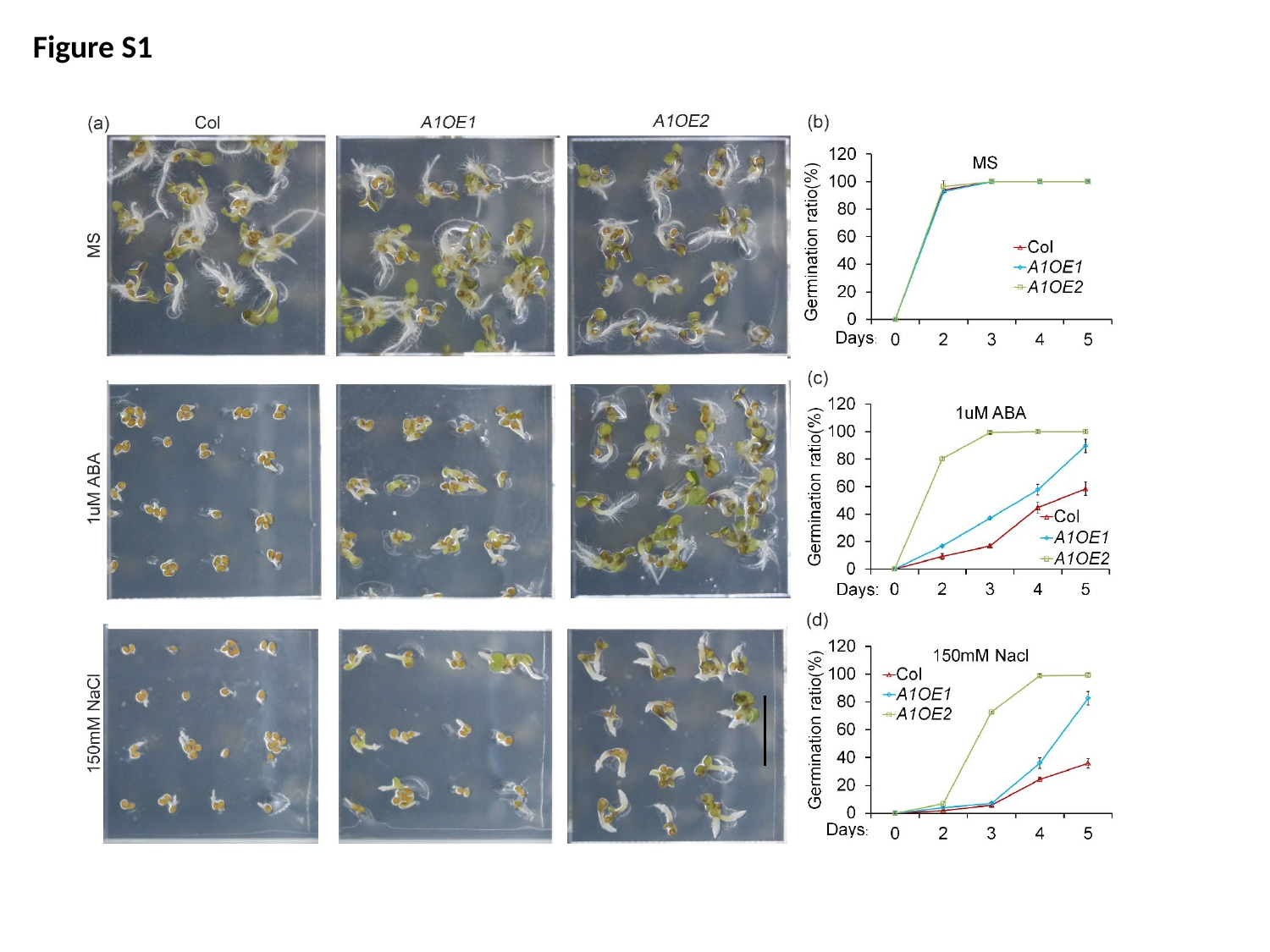

Figure S1

## Slide 2
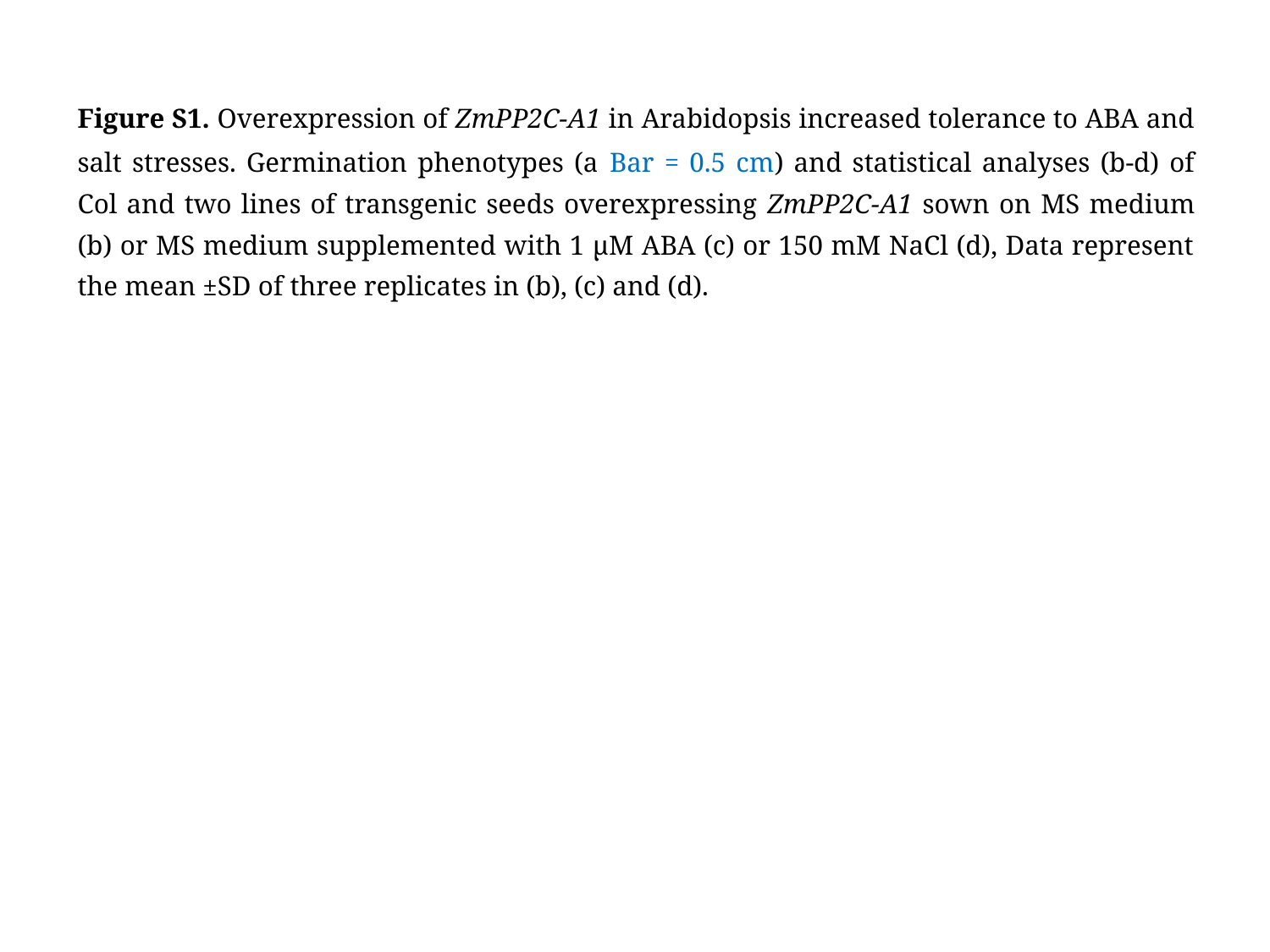

Figure S1. Overexpression of ZmPP2C-A1 in Arabidopsis increased tolerance to ABA and salt stresses. Germination phenotypes (a Bar = 0.5 cm) and statistical analyses (b-d) of Col and two lines of transgenic seeds overexpressing ZmPP2C-A1 sown on MS medium (b) or MS medium supplemented with 1 μM ABA (c) or 150 mM NaCl (d), Data represent the mean ±SD of three replicates in (b), (c) and (d).

## Slide 3
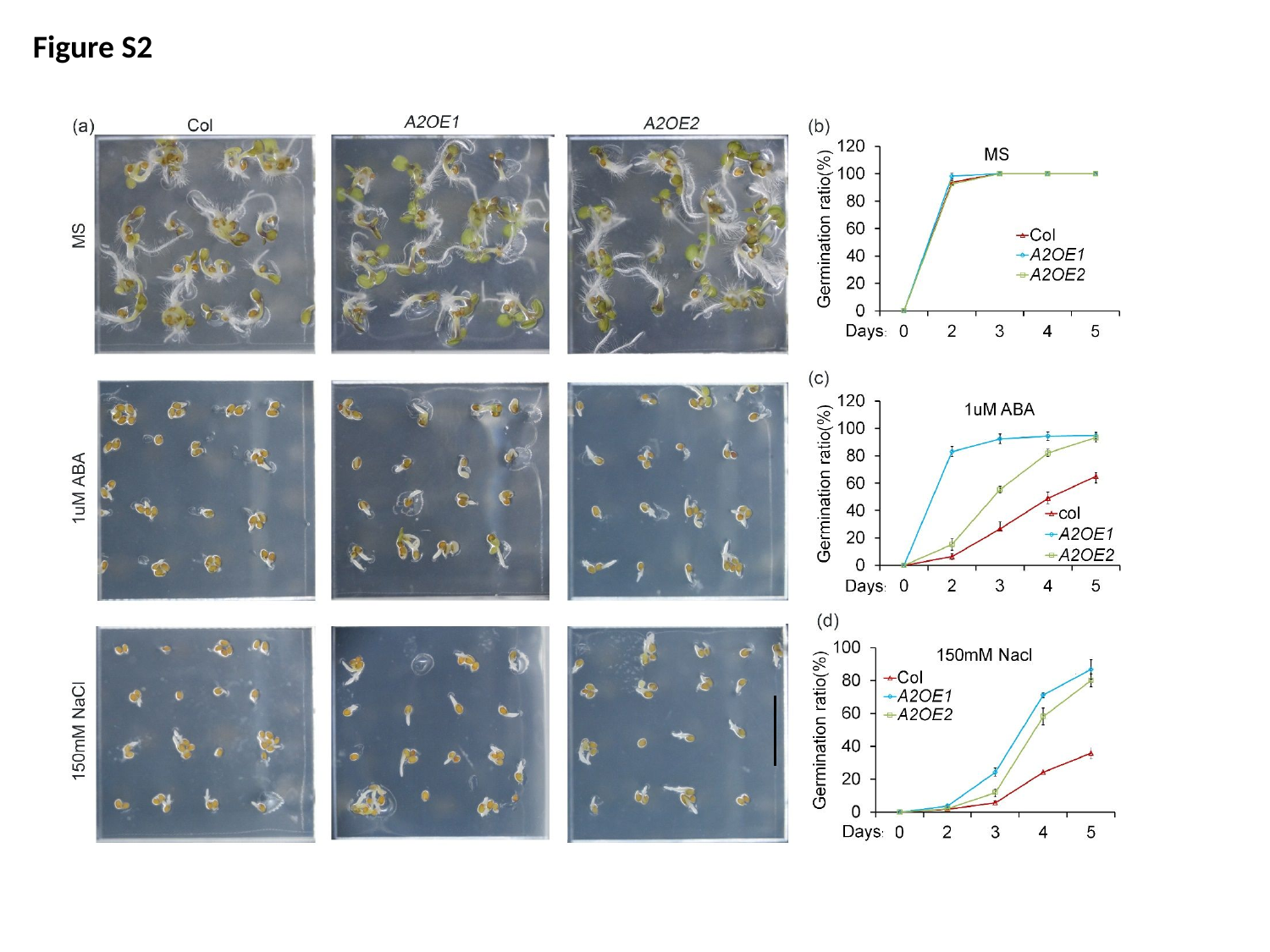

Figure S2

## Slide 4
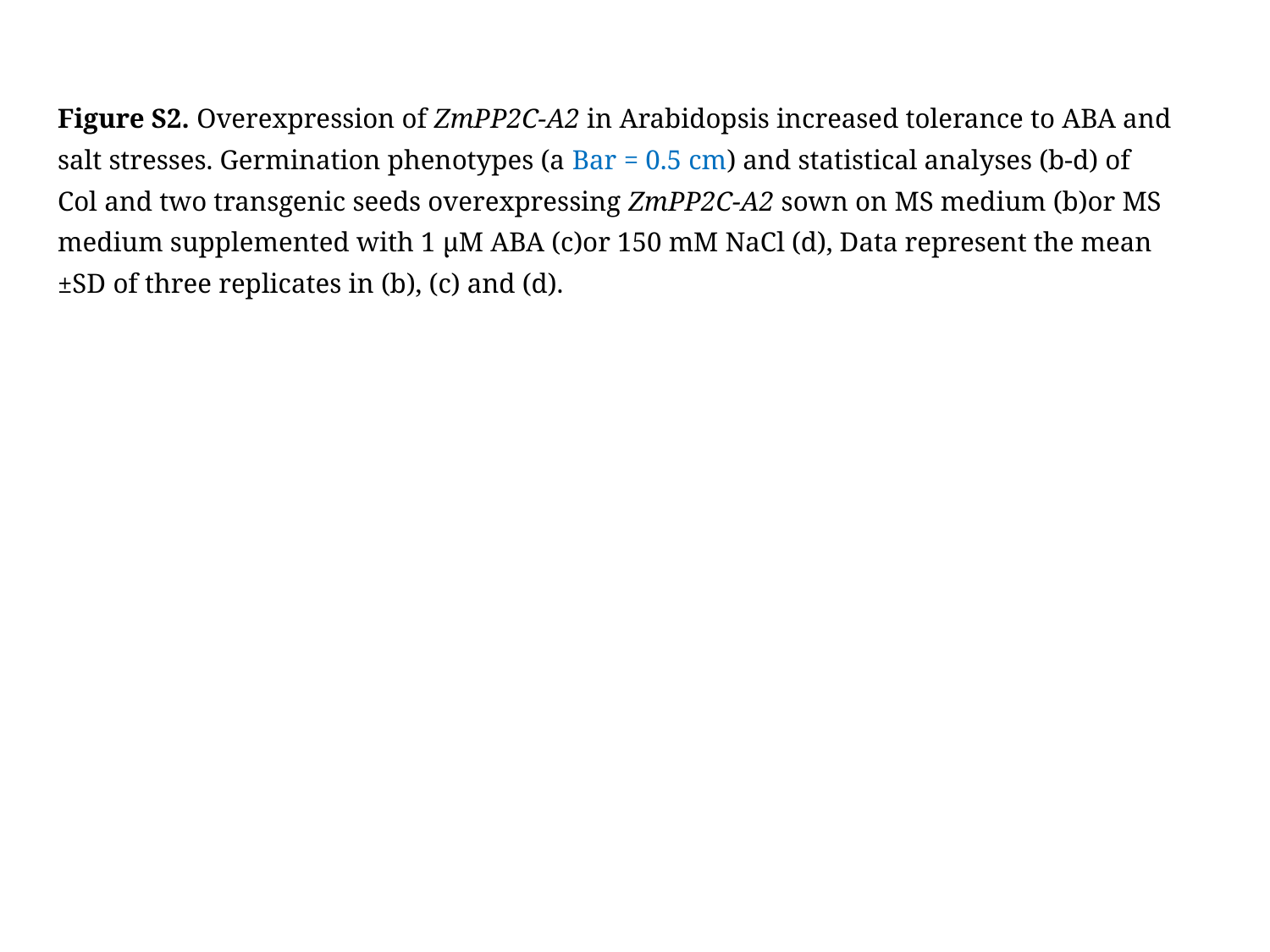

Figure S2. Overexpression of ZmPP2C-A2 in Arabidopsis increased tolerance to ABA and salt stresses. Germination phenotypes (a Bar = 0.5 cm) and statistical analyses (b-d) of Col and two transgenic seeds overexpressing ZmPP2C-A2 sown on MS medium (b)or MS medium supplemented with 1 μM ABA (c)or 150 mM NaCl (d), Data represent the mean ±SD of three replicates in (b), (c) and (d).

## Slide 5
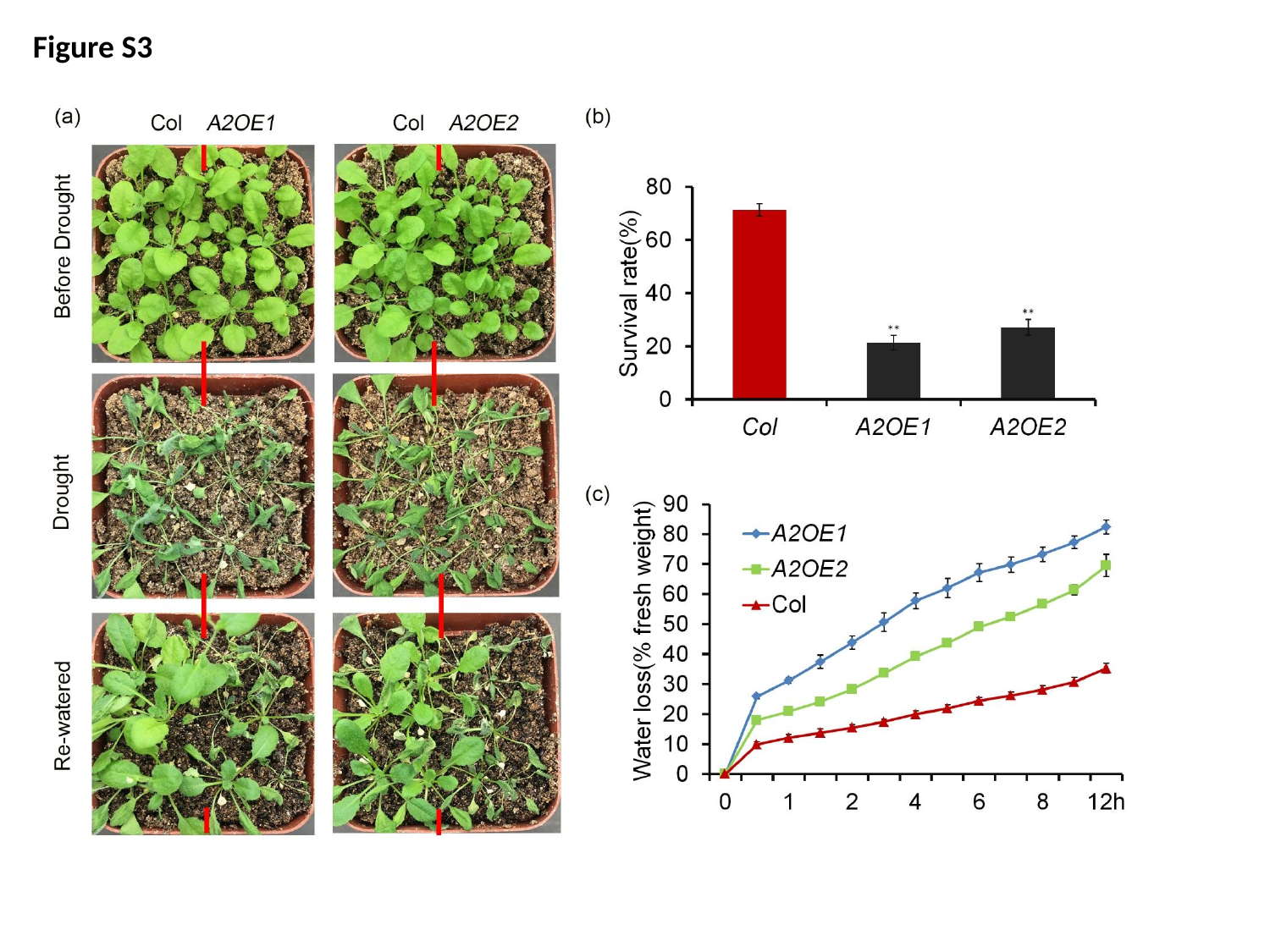

Figure S3

## Slide 6
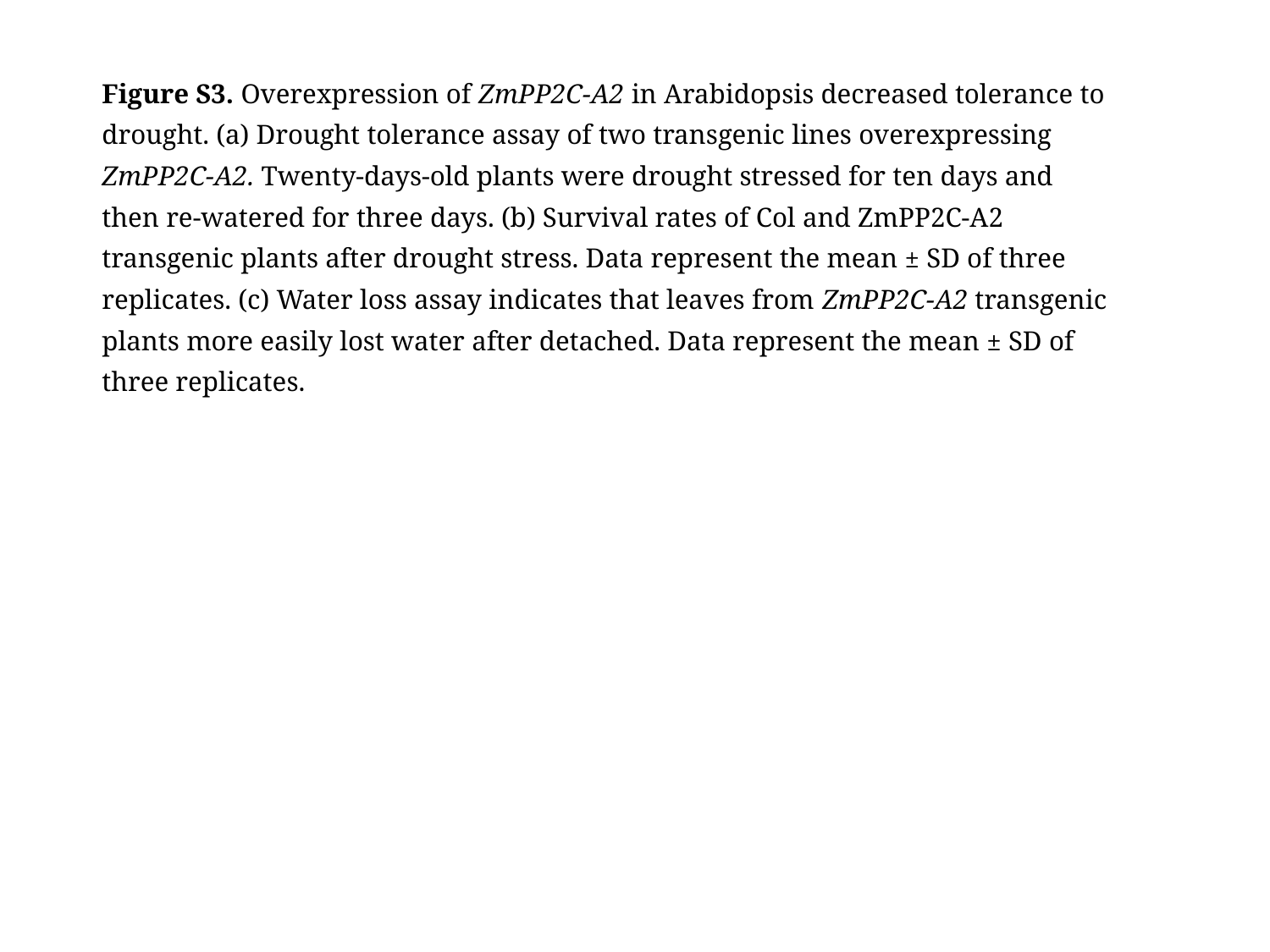

Figure S3. Overexpression of ZmPP2C-A2 in Arabidopsis decreased tolerance to drought. (a) Drought tolerance assay of two transgenic lines overexpressing ZmPP2C-A2. Twenty-days-old plants were drought stressed for ten days and then re-watered for three days. (b) Survival rates of Col and ZmPP2C-A2 transgenic plants after drought stress. Data represent the mean ± SD of three replicates. (c) Water loss assay indicates that leaves from ZmPP2C-A2 transgenic plants more easily lost water after detached. Data represent the mean ± SD of three replicates.

## Slide 7
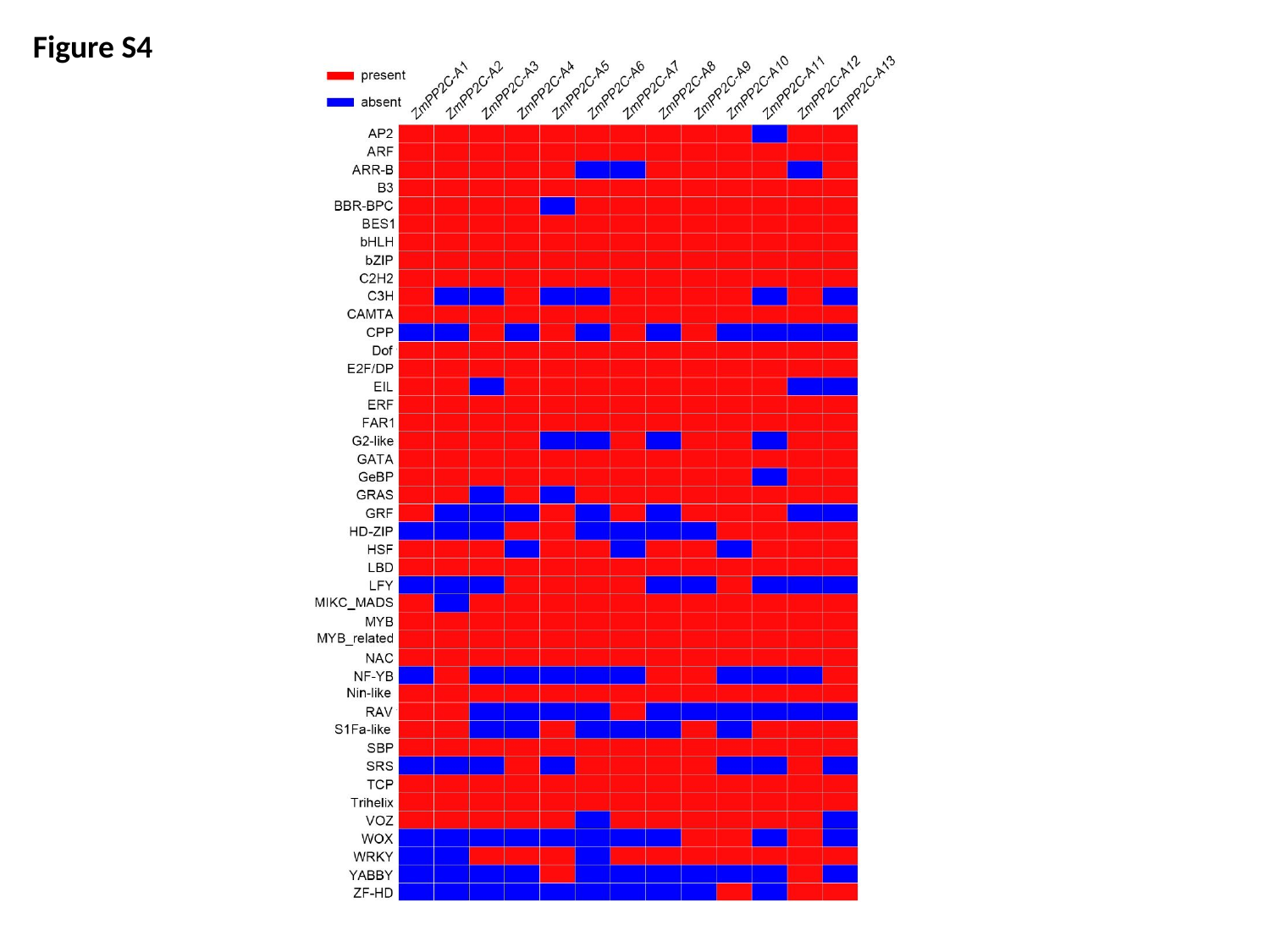

Figure S4

## Slide 8
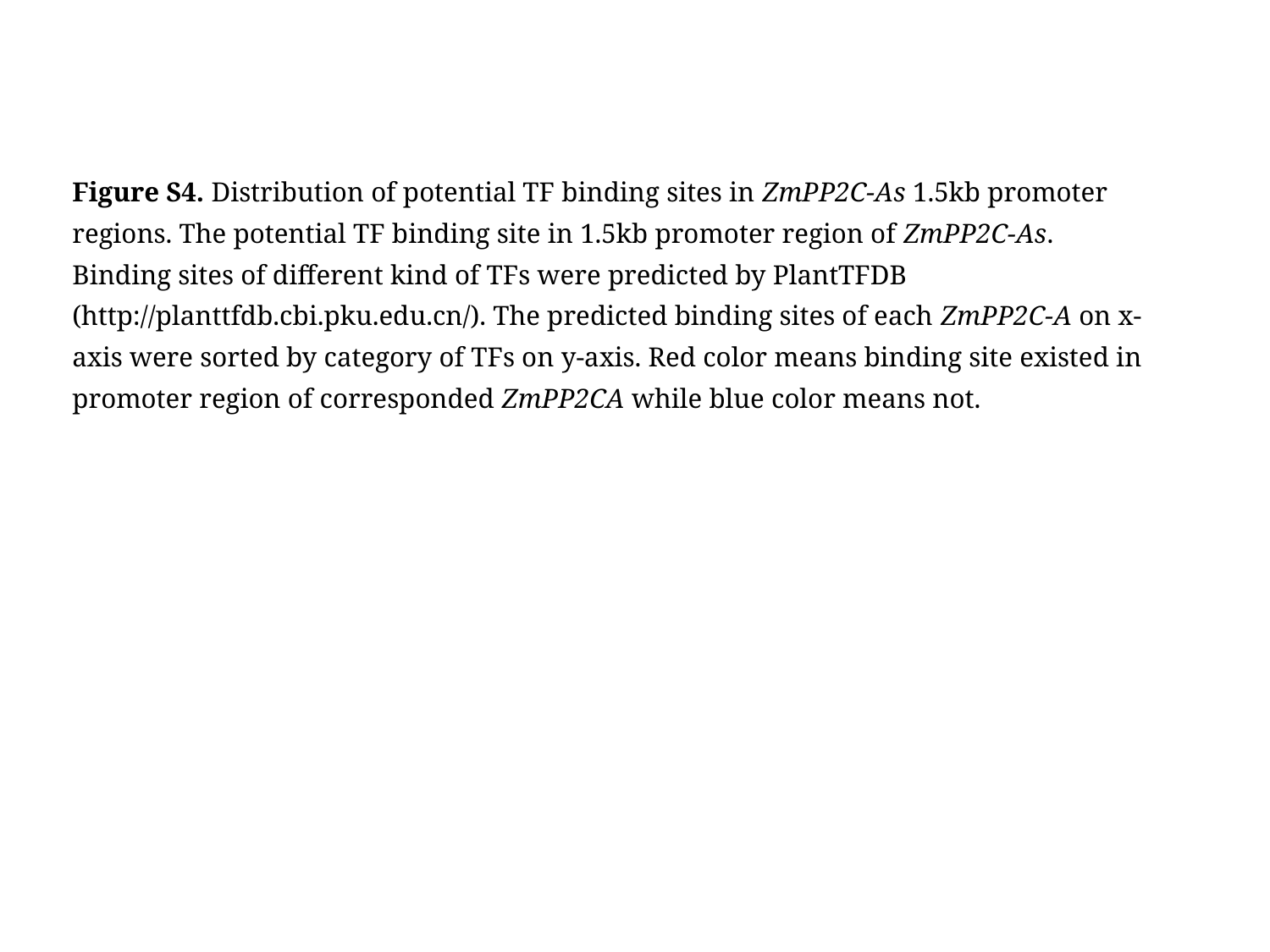

Figure S4. Distribution of potential TF binding sites in ZmPP2C-As 1.5kb promoter regions. The potential TF binding site in 1.5kb promoter region of ZmPP2C-As. Binding sites of different kind of TFs were predicted by PlantTFDB (http://planttfdb.cbi.pku.edu.cn/). The predicted binding sites of each ZmPP2C-A on x-axis were sorted by category of TFs on y-axis. Red color means binding site existed in promoter region of corresponded ZmPP2CA while blue color means not.
