# Supplementary material for: The Maize Clade A PP2C Phosphatases Play Critical Roles in Multiple Abiotic Stress Responses
Source: Int J Mol Sci. 2019 Jul 22;20(14):3573. doi: 10.3390/ijms20143573 (PMC6679055; doi:10.3390/ijms20143573)
Supplement: Supplementary file 1 [file ijms-20-03573-s001.zip › supplementary files of 2019.7.22 ijms 553347/Table S1, Table S2 and Table S3 of ijms.docx]

**Table S1.** Natural variations in *ZmPP2C-A* genes associated with salt resistance in 445 maize inbred lines.

| Gene ID | Name | Function | Polymorphic number* | GLM  (p≤0.01) | GLM+Q  (p≤0.01) | MLM(p≤0.01) |
| --- | --- | --- | --- | --- | --- | --- |
| GRMZM2G059453  GRMZM2G166297  GRMZM2G122228 | *ZmPP2C-A1*  *ZmPP2C-A2*  *ZmPP2C-A6* | NaCl | 44  53  52 | 0  1  0 | 0  0  0 | 0  0  0 |
|  |  | NaCl |  |  |  |  |
|  |  | NaCl |  |  |  |  |

*MAF (Minor Allele Frequency) ≥ 0.05.

**Table S2**. Natural variations in *ZmPP2C-A* genes associated with drought resistance in 368 maize inbred lines.

| Gene ID | Name | Function | Polymorphic number* | GLM  (*P* ≤0.01) | GLM+Q  (*P* ≤0.01) | MLM (*P*≤0.01) |
| --- | --- | --- | --- | --- | --- | --- |
| GRMZM2G059453  GRMZM2G166297  GRMZM2G122228 | *ZmPP2C-A1*  *ZmPP2C-A2*  *ZmPP2C-A6* | Drought | 44  53  52 | 1  2  14 | 0  0  1 | 0  0  1 |
|  |  | ABA Drought |  |  |  |  |
|  |  | ABA Drought |  |  |  |  |

*MAF (Minor Allele Frequency) ≥ 0.05.

**Table S3.** Association analysis in *ZmPP2C-A* genes with maize agronomic traits.

|  | *ZmPP2C-A* | | | | | | | | | | | | |
| --- | --- | --- | --- | --- | --- | --- | --- | --- | --- | --- | --- | --- | --- |
|  | 1 | 2 | 3 | 4 | 5 | 6 | 7 | 8 | 9 | 10 | 11 | 12 | 13 |
| 100 grain weight | ** |  |  |  |  |  |  |  |  |  |  |  |  |
| Cob diameter |  |  | ** |  |  |  | *** |  |  |  | ** |  |  |
| Cob weight |  |  | ** |  |  |  | ** |  |  | ** |  |  |  |
| Ear diameter |  |  |  |  |  |  | *** |  |  |  |  |  |  |
| Ear height |  | ** |  |  |  |  |  | ** |  | ** | ** |  |  |
| Ear leaf length |  |  |  | ** |  | ** |  |  |  | ** | ** |  |  |
| Ear leaf width |  |  |  |  |  |  | *** |  |  |  |  |  |  |
| Ear length | ** | ** |  |  |  |  |  |  |  | ** | ** |  |  |
| Heading date | ** |  |  | ** |  | ** |  | ** |  | ** |  |  |  |
| Kernel width |  |  |  |  |  | ** | ** |  |  | ** | ** |  |  |
| Kerner number per row |  |  |  |  |  |  |  |  |  |  |  |  |  |
| Leaf number above ear |  | ** |  |  | ** | ** | ** |  |  | ** | ** |  |  |
| Plant height |  |  |  |  | ** | ** |  | *** |  | ** | ** |  |  |
| Pollen shed |  |  |  | ** |  | ** |  | ** |  |  | ** |  |  |
| Silking time |  | ** |  | ** |  |  |  | ** | ** |  |  |  |  |
| Tassel branch number | ** | ** |  | ** |  |  |  |  |  | ** | ** |  |  |
| Tassel main axis length |  |  |  | ** | ** |  |  |  |  | ** | ** |  |  |

MLM model was adopted，** stands for *P* ≤ 0.01, *** stands for *P* ≤ 0.001
