# Supplementary figures and images for: The Maize Clade A PP2C Phosphatases Play Critical Roles in Multiple Abiotic Stress Responses
Source: Int J Mol Sci. 2019 Jul 22;20(14):3573. doi: 10.3390/ijms20143573 (PMC6679055; doi:10.3390/ijms20143573)

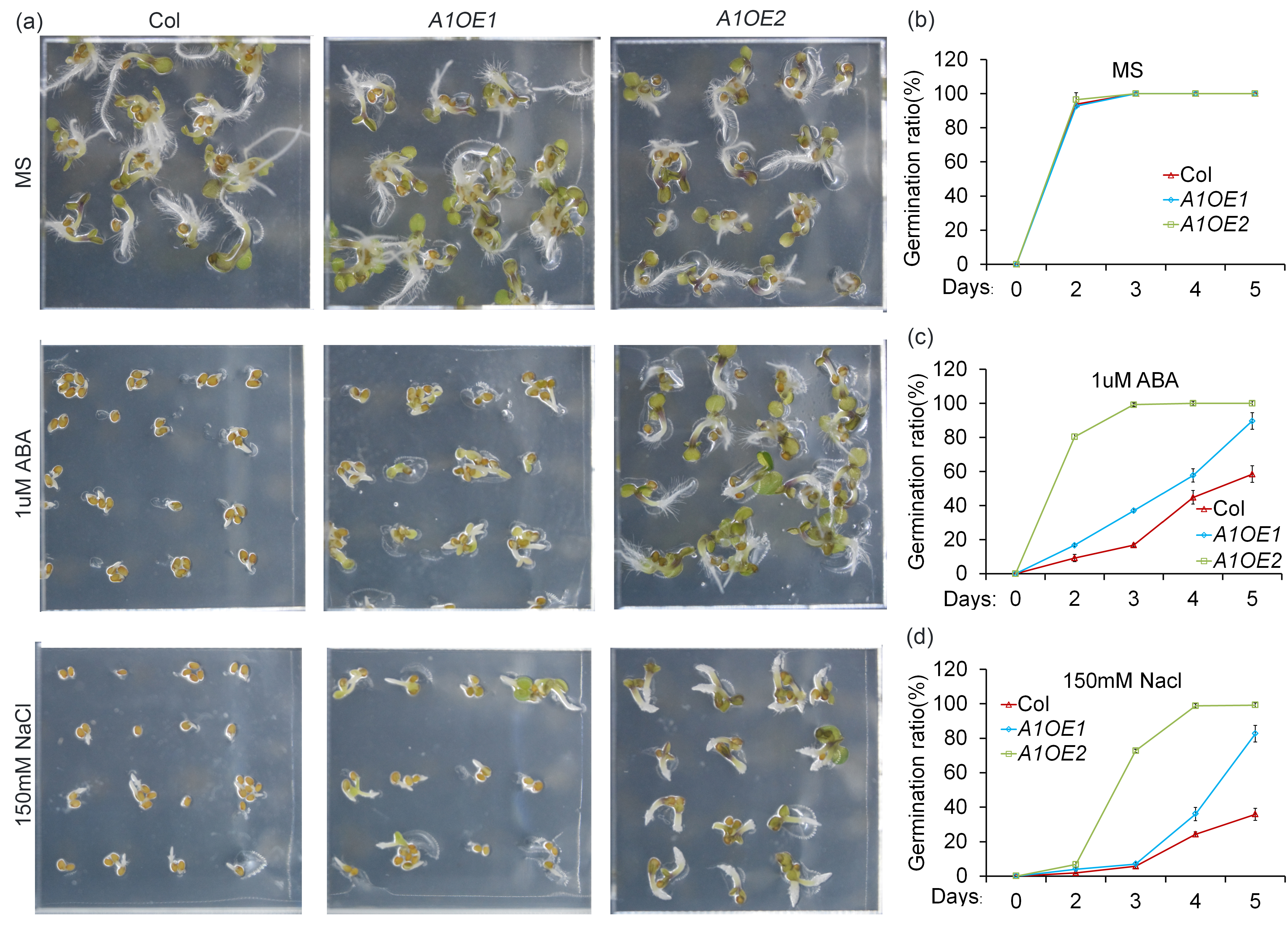

Supplement: Supplementary file 1 [file ijms-20-03573-s001.zip › supplementary files of 2019.7.22 ijms 553347/Figure S1 of ijms.jpg]

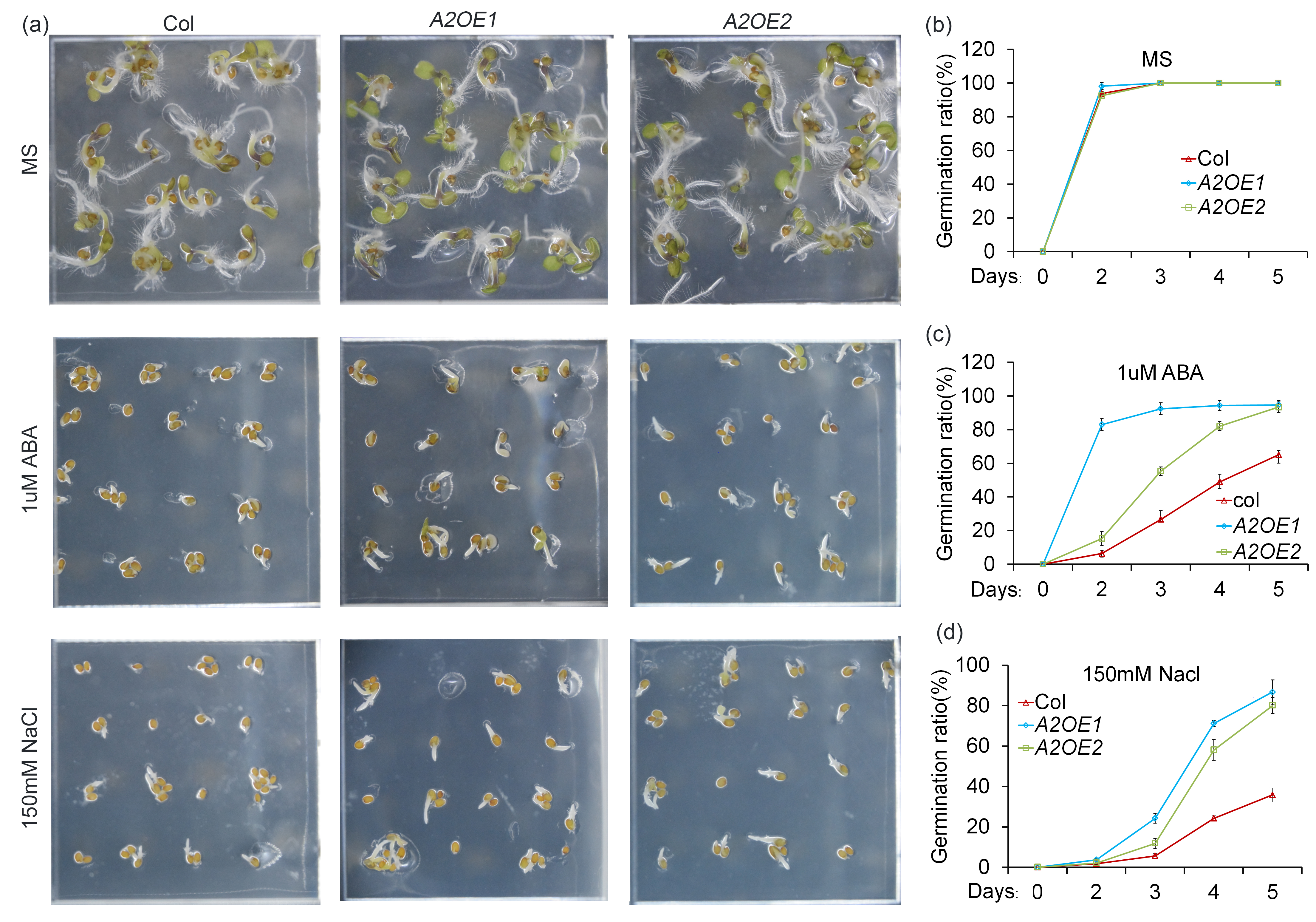

Supplement: Supplementary file 1 [file ijms-20-03573-s001.zip › supplementary files of 2019.7.22 ijms 553347/Figure S2 of ijms.jpg]

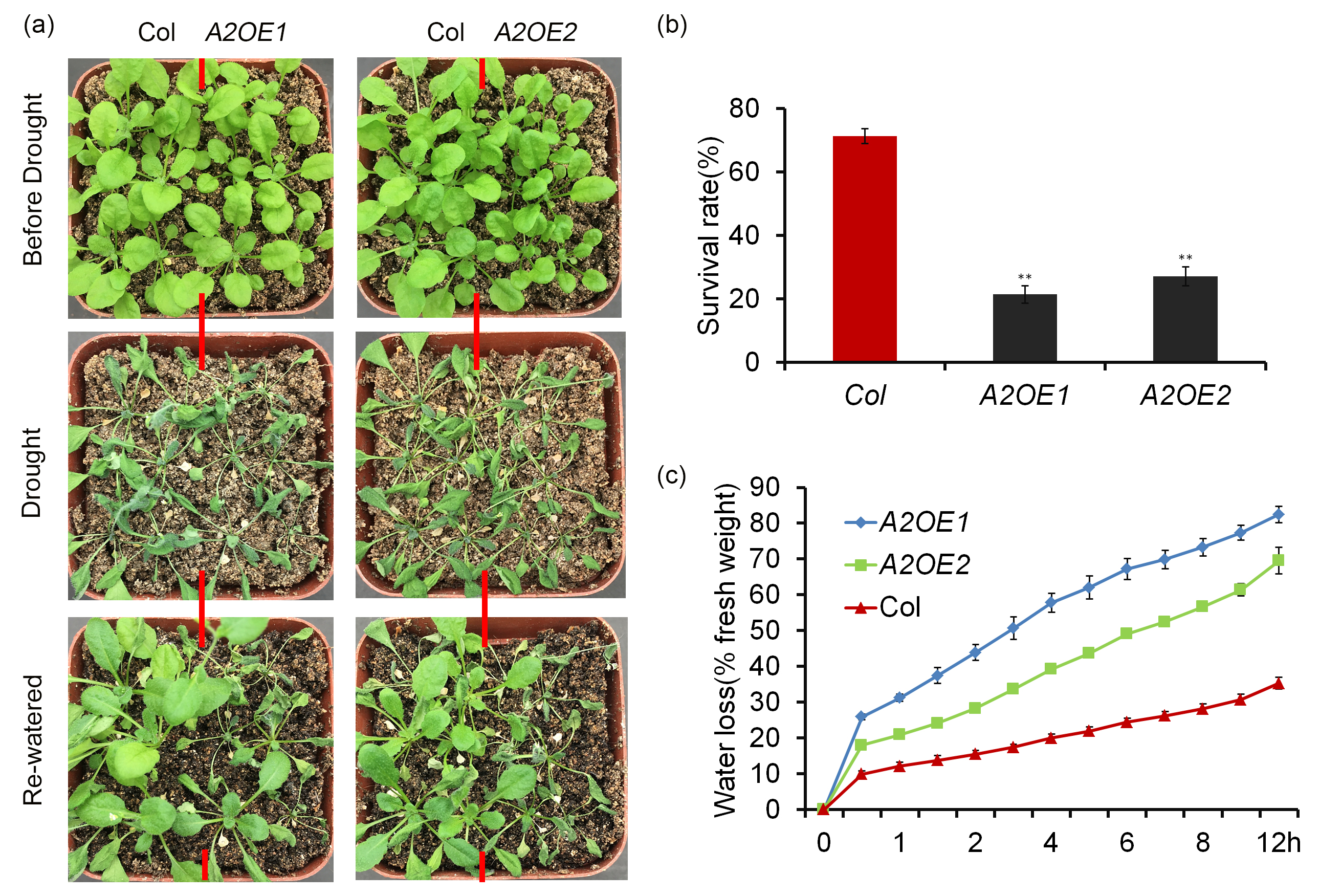

Supplement: Supplementary file 1 [file ijms-20-03573-s001.zip › supplementary files of 2019.7.22 ijms 553347/Figure S3 of ijms.jpg]

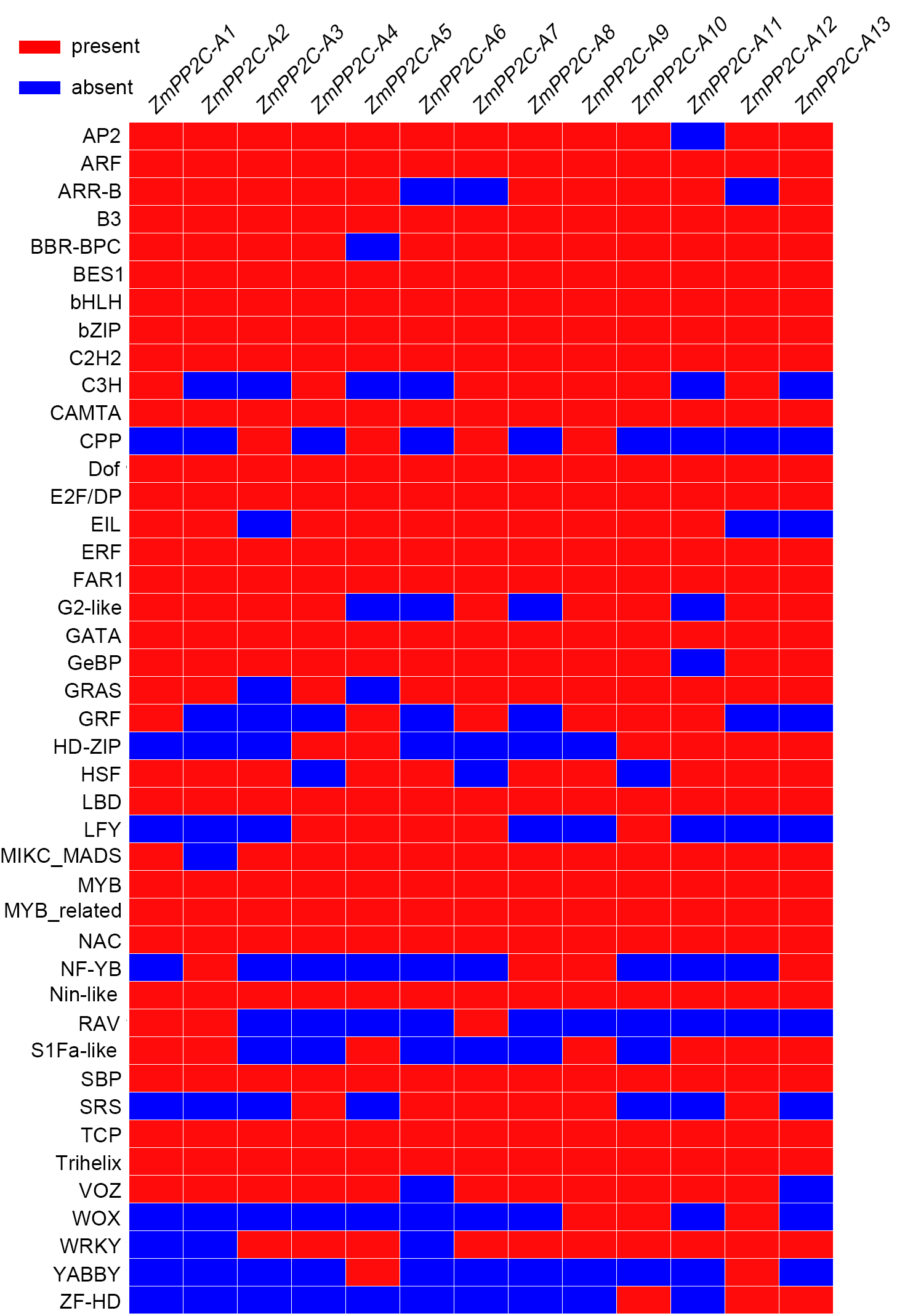

Supplement: Supplementary file 1 [file ijms-20-03573-s001.zip › supplementary files of 2019.7.22 ijms 553347/Figure S4 of ijms.jpg]
